# A case study of sudden-onset cortically mediated visual impairments in a 12-year-old

**DOI:** 10.21203/rs.3.rs-7861568/v1

**Published:** 2025-10-17

**Authors:** Jan W. Kurzawski, Robert G. Alexander, Nicolas Brunet, Orrin Devinsky, Fengping Hu, Stephen L. Macknik, Susana Martinez-Conde, Molla Nawsher, Bas Rokers, Timothy M. Shepherd, Ashwin Venkatakrishnan, Jonathan Winawer

**Affiliations:** 1 New York University, Department of Psychology, New York, NY, USA; 2 Faculty of Psychology and Neuroscience, Maastricht University, Maastricht, Netherlands; 3 New York Institute of Technology, Department of Psychology & Counseling, New York, NY, USA; 4 California State University, San Bernardino, Department of Psychology, San Bernardino, CA, USA; M New York University School of Medicine, Department of Radiology, New York, NY, USA; X New York University School of Medicine, Departments of Neurology, Neurosurgery, and Psychiatry, New York, NY, USA; Y Psychology, New York University Abu Dhabi, Abu Dhabi, United Arab Emirates; Z State University of New York Downstate Health Sciences University, Departments of Ophthalmology, Neurology, and Pharmacology & Physiology, Brooklyn, NY, USA

**Keywords:** akinetopsia, deficits in clutter, visual crowding, focal cortical dysplasia

## Abstract

Strokes and blunt trauma can cause large changes in perception. It is rare, however, to have a sudden but persistent change to perception in the absence of trauma. Here we report a case of a 12-year-old male who reported a sudden-onset change in vision without any trauma, with akinetopsia-like symptoms: an inability to see motion. In contrast to classical cases of akinetopsia, informal testing revealed normal motion perception for simple stimuli, but difficulty in recognition of moving objects in visual clutter. Psychophysical testing confirmed normal random dot motion sensitivity and a large deficit in object recognition in clutter in moving displays, and surprisingly, in static displays. Oculomotor behavior showed both slowed saccades and difficulty in smooth pursuit. Anatomical and functional MRI showed largely intact retinotopic maps and robust responses to visual motion, including in canonical cortical motion processing areas. However, MRI imaging revealed a right lingual gyrus gray-white contrast blurring with corresponding severe focal FDG hypometabolism on PET, consistent with focal cortical dysplasia (FCD). We speculate that the abnormality in ventral cortex affects recognition in clutter, which manifests as a subjective experience of akinetopsia-like symptoms, especially in complex dynamic scenes.

## 1. Introduction

The human visual system is a complex network of interconnected brain regions, each playing a crucial role in the perception and interpretation of our surroundings. Disruptions within this network, such as those caused by strokes or traumatic brain injuries, can lead to significant and often debilitating perceptual deficits (Sacks, 2010; Semir Zeki, 1993). Depending on the site of the lesion, the perceptual effects can be quite specific. For example, akinetopsia is a selective impairment in motion perception, typically associated with lesions in the motion processing areas of the brain, particularly the middle temporal visual area (MT/V5) and surrounding regions (S. Zeki, 1991; Zihl et al., 1983). Akinetopsia presents as a profound inability to perceive continuous motion, often described by patients as seeing the world in a series of static snapshots.

However, the clinical manifestation of motion perception deficits can be nuanced and a variety of underlying neurological conditions may be associated with reports of akinetopsia-like symptoms (Browne et al., 2024; Horton & Trobe, 1999; Van Swol et al., 2024). This is particularly true in cases of sudden-onset, persistent visual impairments with no immediately obvious neurological cause. Here, we present a case study of a 12-year-old male who reported a sudden and significant change in his visual perception, including akinetopsia-like symptoms, despite the absence of any history of trauma or neurological disorders. The patient described a profound difficulty in perceiving moving objects, particularly in cluttered environments, leading to substantial functional impairment in daily activities. This presentation challenged the initial hypothesis of a typical motion-related cortical dysfunction, as standard neurological examinations and initial MRI imaging yielded unremarkable findings.

The discrepancy between the patient’s subjective symptoms and standard neurological evaluations prompted a comprehensive investigation, including detailed psychophysical testing, advanced neuroimaging techniques, and oculomotor assessments. We hypothesized that the patient’s symptoms might stem from a more subtle, potentially localized, cortical abnormality affecting higher-level visual processing, rather than a primary deficit in motion perception. Specifically, following a series of informal tests of visual perception, we considered that deficits in object recognition within cluttered visual scenes might manifest subjectively as akinetopsia. Clutter and crowding effects, where object recognition is impaired by surrounding stimuli, are well-documented phenomena (Whitney & Levi, 2011). Object recognition involves the ventral visual stream and particularly the visual areas along the ventral surface of the occipital and posterior temporal lobes (Grill-Spector et al., 1998; Kanwisher et al., 1997), and we speculated that an abnormality in this processing stream could lead to the patient’s reported difficulties.

This report details the patient’s clinical presentation, the comprehensive battery of tests performed, and the surprising findings that suggest a novel interpretation of akinetopsia-like symptoms in the context of focal cortical dysplasia and deficits in object recognition within clutter. By investigating this unique case, we aim to shed light on the complex interplay between motion perception, object recognition, and higher-level visual processing, and to provide insights into the neural mechanisms underlying sudden-onset visual impairments in pediatric patients.

## 2. Results

### 2.1. Case Description

A 12-year-old right-handed male with no past medical or neurological disorders, suddenly developed visual changes in 2022, which he noticed one evening when he was playing a game with his sister. When he went to sleep that night he told his father his vision was blurry. The next morning, he went to the school nurse reporting that “Kids would appear in front of me or right next to me but I did not see them coming. Walking in the hall people would just appear next to me suddenly.”

He initially had difficulty walking up or down stairs as his vision disoriented him. When he looked at his feet, he was more disoriented. By the time of a neurological exam by author O.D. two months later, the patient had accommodated to some of the problems, but the problems had not subsided. For example, he could play four squares by using the angle of his dad’s hand and the noise of the ball bouncing to guess the location of the ball. He still could not play catch with a ball as the ball just suddenly appeared inches from him, and his hand could not adjust in time. If a ball was thrown to his side, he would first see it when it crossed the plane of his eyes. If a ball was thrown quickly to his side he might not see it at all. Sudden movements on screen were hard to follow, such as a movie with a chase scene. He had great difficulty walking in a crowded place like Manhattan. In follow up over the next two years, symptoms varied in degree and intensity but he adjusted both physiologically and emotionally. However, the symptoms persisted relatively unchanged.

An initial MRI with gadolinium and routine EEG were normal. A 48-hour ambulatory EEG was normal. Routine labs, as well as thyroid function studies, erythrocyte sedimentation rate, C-reactive protein, and autoimmune studies were all normal.

Prior to the neurological exam by O.D., the patient underwent two neuro-ophthalmological evaluations. Dilated fundoscopic slit lamp examination, optical coherence tomography, visual acuity, extra-ocular movements (full range without nystagmus, vestibulo-ocular reflex), color vision (8/8 Ishihara color plates in each eye), stereoscopic vision and functional vergence and convergence assessments were normal. One found a normal visual field while the other noted the examination was ‘unreliable’ but Humphrey visual field testing in the right eye showed mean deviation score of −3.98 dB and superior nasal depression with normal foveal threshold. Testing in the left eye showed mean deviation score of −9.76 dB and superior and inferior temporal depression with subnormal foveal threshold. These results were interpreted as: abnormal visual field in both eyes, nonspecific. Repeat testing in the left eye was more reliable and showed mean deviation score of −4.97 dB and inferior temporal depression. Eye movements were reported normal except that “there were a few saccadic intrusions during pursuit testing.”

An FDG integrated PET/MRI in 4 months after the initial event revealed a subtle posterior right lingual gyrus cortical signal abnormality with corresponding severe focal FDG hypometabolism, suggestive of focal cortical dysplasia. There was also symmetric moderate bilateral occipital and medial temporal lobe FDG hypometabolism consistent with a temporal occipital seizure network (he never had a clinical seizure or epileptiform activity on EEG).

### 2.2. Informal testing

We initially examined visual function informally by showing the patient demonstrations from an undergraduate lecture on visual motion. His interpretations of most of the demonstrations were typical with a few exceptions. This testing was conducted to generate hypotheses for subsequent quantitative evaluation.

He successfully interpreted gratings and plaid motion, high coherence moving dot displays, noiseless point light animation displays, and motion without movement demonstrations. He had typical responses to reverse phi, motion aftereffect and apparent motion demonstrations. He had difficulty interpreting a point light walker with noise (additional dots with random motion), and with recognizing moving objects in clutter. See **Supplemental methods 1** for details.

The patient also underwent informal testing at SUNY Downstate 4, 9, and 15 months after the patient’s initial report in 2022. He successfully interpreted implied motion photographs of people engaged in sports and other physical activities. He reported perception of illusory motion from static repetitive patterns, such as from the rotating snakes illusion, consistent with typical observers (Otero-Millan et al., 2012). He reported difficulty visualizing fluid, non-stuttering motion (i.e. remembering or imagining the motion of a bird flying across the sky). He was able to read physical and electronic (static) texts.

### 2.3. Random dot motion perception

To study low level motion processing quantitatively, we assessed the patient’s performance in motion direction discrimination using random dot kinematograms. We asked the patient to judge motion direction as we varied the speed, duration and coherence of dots in three experimental conditions. We conducted the same experiment with an age- and sex-matched control (“C1”). Overall, the patient showed no clear deficits in any of these tasks.

#### 2.3.1 Results

##### Stimulus speed

**Figure 1.**
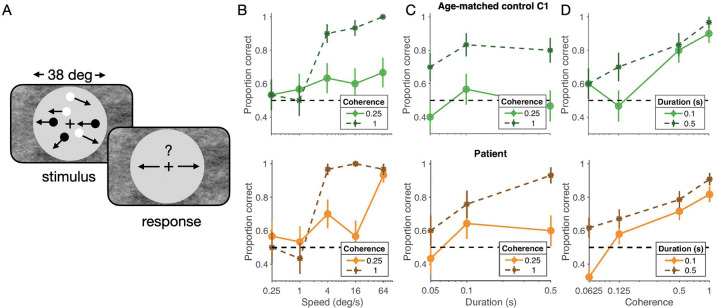
Motion discrimination task and performance. Participants judged motion direction (left/right) of a large (36 deg diameter) field of moving dots (A), while we varied motion speed (B), coherence (C), and presentation time (D). Results were similar for the patient (lower panels) and C1, an age-matched control (upper panels). The data are plotted as means +/− 68% confidence interval of the binomial estimates.

At full dot coherence (100%), motion discrimination was near ceiling for high speeds (> 4 deg/s) but fell to chance for low speeds (<= 1 deg/s) for both patient and control subject (Figure 1A, solid lines). At low dot coherence (25%), higher speeds again had a positive, albeit much smaller, effect on accuracy (Figure 1A, dashed lines). Critically, these patterns were the same for the patient and an age-matched control (C1), and broadly consistent with data from adult observers (De Bruyn & Orban, 1988; D. W. Williams & Sekuler, 1984).

##### Stimulus duration

Next, we fixed stimulus speed at 8 deg/s (within the range of speeds where full coherence stimuli produced near-ceiling performance) and varied stimulus duration. We reasoned that if there was a deficit in the temporal integration of motion signals, we would see flat performance as a function of stimulus presentation time. We did not observe this. Both the patient and control showed an increase in accuracy from 50-ms to 100-ms durations (Figure 1B).

##### Stimulus coherence

Finally, we held stimulus speed constant and varied stimulus coherence between 6.25 and 100% for two stimulus durations (100 and 500 ms). Here we reasoned that if the patient had difficulty recognizing motion signals in noise, they might show abnormally low performance under high noise (i.e. low coherence) conditions. However, we found similar performance for the patient and control. Performance monotonically decreased for lower coherence conditions, but like the control remained above chance even at 6.25% coherence, provided stimulus presentation time was sufficiently long (Figure 1C).

Taken together, this pattern of results suggests that the detection of instantaneous motion signals, the integration of motion signals over time, and the ability to detect coherent motion signals in the presence of noise, were normal. While we cannot rule out the possibility of a subtle low-level motion impairment, the similarity to the control means there is no large deficit. This contrasts with substantial motion perception deficits associated with lesions to the MT cortical regions (Zihl et al., 1983) and what might be expected based on a diagnosis of classical akinetopsia-like symptoms.

### 2.4. Recognition in clutter

Given the unremarkable results in dot motion discrimination and the pattern of perceptual failures in our informal testing, we hypothesized that the patient’s perceptual difficulties stemmed from impaired object identification in clutter rather than deficits in low-level motion perception. To test this, we designed an experiment to test recognition in clutter. In one condition, a moving stimulus was presented among stationary distractors. We tested the patient and the same age- and sex-matched control as the motion discrimination experiment (C1).

**Figure 2.**
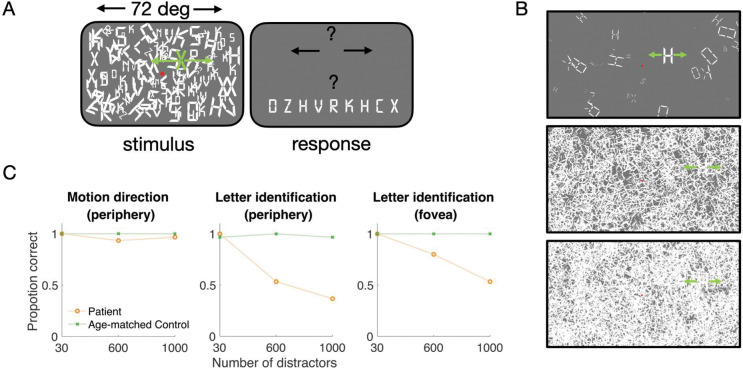
Letter identification in noise. (A) Each trial began with central fixation (red dot) and an array of letters for 1 to 2 seconds. For the peripheral task, one letter in the array then moved leftward or rightward. The participant was asked the direction of motion and the letter identity. For the foveal task, after the 1 to 2 second static array, the central letter (B) Distractor number was either 30, 600, or 1000. (C) Proportion correct for motion direction (2AFC) in the peripheral task for the patient (orange) and controls (blue, green). (D) Proportion correct for letter recognition in the peripheral task (same experiment as panel A). (E) Proportion correct in a separate experiment in which the target letter was presented at the fovea and remained stationary, and all other letters moved either left or right.

In the peripheral experiment, participants fixated centrally while viewing an array of stationary letters of variable density (Figure 2A,B). After one to two seconds of fixation, one of the letters began translating either leftward or rightward (4 deg/s, for one second) while the remaining letters stayed stationary, at which point the participants could move their eyes. They were then asked to report first the direction of motion and then the letter identity. Both the patient and control were at or near ceiling in motion direction. However, the patient’s performance on letter identity declined as clutter increased, whereas the control participant remained at ceiling (Figure 2C).

While the patient tended to make eye movements toward the target letter, the saccade latency was greater than both controls, and the accuracy was lower. To disentangle the oculomotor and perceptual effects, we conducted a second experiment with a stationary central target. Here, the participants again viewed a stationary letter array for one to two seconds. In this experiment, however, the central target letter remained stationary and the other letters moved leftward or rightward at 4 deg/s. Participants were asked to identify the central, stationary letter. The control subject was again near ceiling whereas the patient was impaired at high densities. (Figure 2C, rightmost panels). The results from the two experiments suggest impairments in object identification within clutter, and intact motion perception.

**Figure 3.**
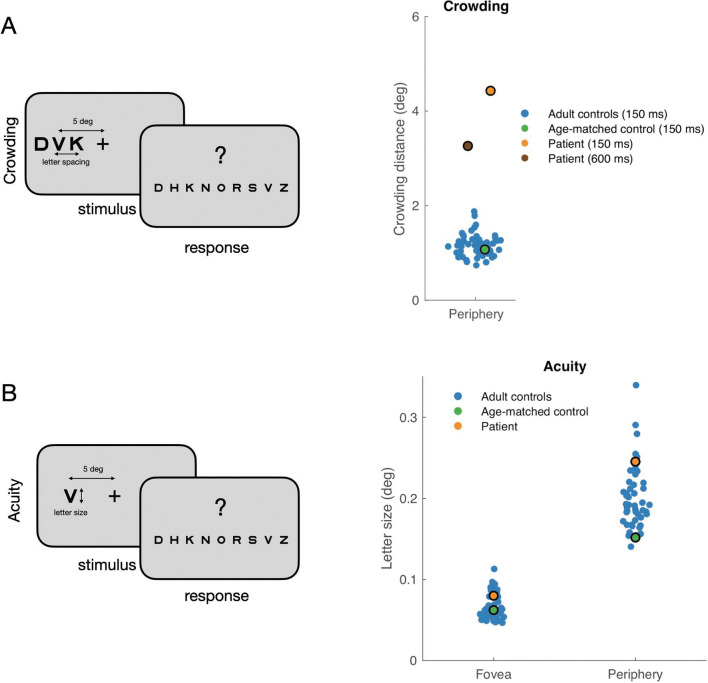
Size and spacing limits of letter recognition. (A) In the crowding experiment, the participant identifies the middle letter of a trigram, centered at 5 deg to the left or right of fixation. A psychophysical staircase varies the spacing between letters across the experiment. The crowding distance is the minimal center-to-center letter spacing between a target letter and flankers at which letter accuracy reaches criterion performance. Crowding distance is plotted for young adult control participants (18 to 21, blue dots), for an age and sex-matched control (green dot) and for the patient. (B) For the acuity experiment, the participant identifies an isolated letter at 5 deg to the left or right of fixation. The psychophysical staircase varies the size of the letter. We plot letter size as the smallest size at which the participant reaches criterion accuracy. Adult control data are replotted from Kurzawski et al (2023).

To investigate whether a deficit in cluttered recognition persisted in the absence of any motion, we measured the patient’s “crowding” threshold, the minimum spacing between a peripheral target letter and surrounding letters needed to recognize the target (Figure 3A). The patient’s crowding thresholds were well outside the range of typical young adults (18–21 yo) and ~4 times higher than their mean: 4.4 deg and 3.9 deg for test-retest, vs 1.16 ± 0.24 deg (mean±SD) of the typical population, indicating that letter recognition in the absence of any visual motion is also impaired. The high crowding thresholds for the patient were similar on the left and right (4.3 vs 4.5 deg, L vs R, averaged across test-retest). The crowding threshold for an age- and sex-matched control, C2, was inside the young adult distribution, as expected from developmental measurements of crowding (Waugh et al., 2018). To assess whether the elevated threshold was due to the relatively brief stimulus presentation (150 ms), we measured the patient’s crowding threshold with a 600-ms duration. Even with the duration increased 4-fold, the crowding distance was still well outside the range of typical measurements. Finally, to assess the specificity of the recognition deficit, we measured letter acuity in the periphery (±5 deg) and fovea. Both thresholds were within the normal range (Figure 3B). The patient’s peripheral acuity was 0.24 deg (mean±SD of controls: 0.20 ± 0.04) and foveal acuity was 0.08 deg (mean±SD of controls: 0.065 ± 0.01).

Taken together the results from the dynamic and static clutter experiments suggest that, rather than a deficit in motion perception, the perceptual difficulties experienced by the patient are better characterized as an impairment in recognition in clutter, which applies in both dynamic and static displays.

### 2.5. Oculomotor Findings

To identify any potential oculomotor deficits, we assessed the patient’s performance on a comprehensive battery consisting of eight oculomotor tasks. The data revealed significant deficits, especially in performance on smooth pursuit tasks. The overall oculomotor results point towards an overall sluggishness, with slower eye movement speeds—during both smooth pursuit and saccades—and reduced visual exploration of natural scenes. The battery revealed a more severe oculomotor impairment than initially ascertained from clinical observation.

#### 2.5.1 Smooth pursuit

**Figure 4.**
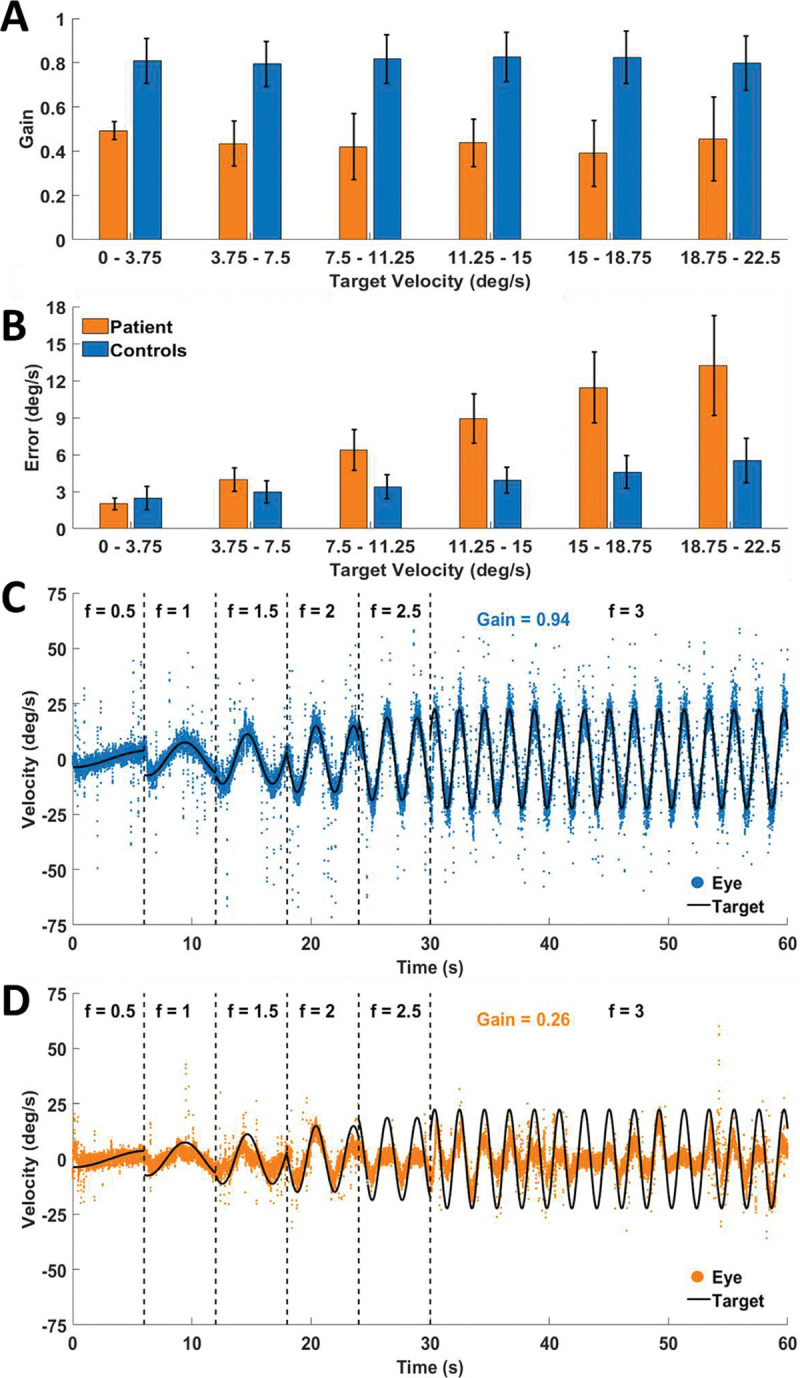
Smooth pursuit. Gain **(A)** and error **(B)** for the patient (orange; averaged across all three recording sessions) and for control participants (blue). For control participants, error bars show the standard deviation between the participants. For the patient, error bars show standard deviation between recording sessions. Representative sample trials from the smooth pursuit task, for the patient (**C**) and a control participant, age 25 (**D**). The target velocity is indicated in black, and the gaze velocity in green. The patient’s performance is deteriorated as compared to that of the control participant.

The patient experienced substantial difficulties attempting to track a target in the smooth pursuit task (see Supplemental Movies 1–4). The patient’s smooth pursuit gain (i.e. the ratio between the mean velocities of eye and target motion) was lower than that of control participants (Figure 4A). Pursuit error (the difference between the speed of the eye and the target was also higher for the patient than for control participants, with the difference increasing as a function of target speed (Figure 4B).

Permutation-based significance testing was used to evaluate whether the patient’s smooth pursuit performance (from three sessions) differed significantly from controls. For pursuit gain and error, we computed the patient’s observed mean and compared it to a null distribution generated by randomly sampling three control values (without replacement) from the full set of controls (N = 33), repeating this process 10,000 times. On each iteration, we calculated the mean of the sampled controls. A two-sided p-value was then computed as the proportion of permuted means whose absolute deviation from the control group mean was greater than or equal to the absolute deviation of the patient’s mean from the control mean. This approach makes no assumptions about distributional normality and provides a robust, non-parametric test of differences. This permutation-based test confirmed that pursuit performance differed between the patient and controls across all measured metrics. These differences held for the different ages of control participants, including aged-matched peers (see **SI Figure 1**). All of the bootstrapping values for each target speed bin were significantly different for target speed, gain, and error, except for the two slowest bins (where error was not significantly different between the patient and controls). The reduced gain and increased error can be seen by comparing representative smooth pursuit trials for the patient (Figure 4C) and a control participant, aged 12 (Figure 4B).

#### 2.5.2 Saccadic Main Sequence

**Figure 5.**
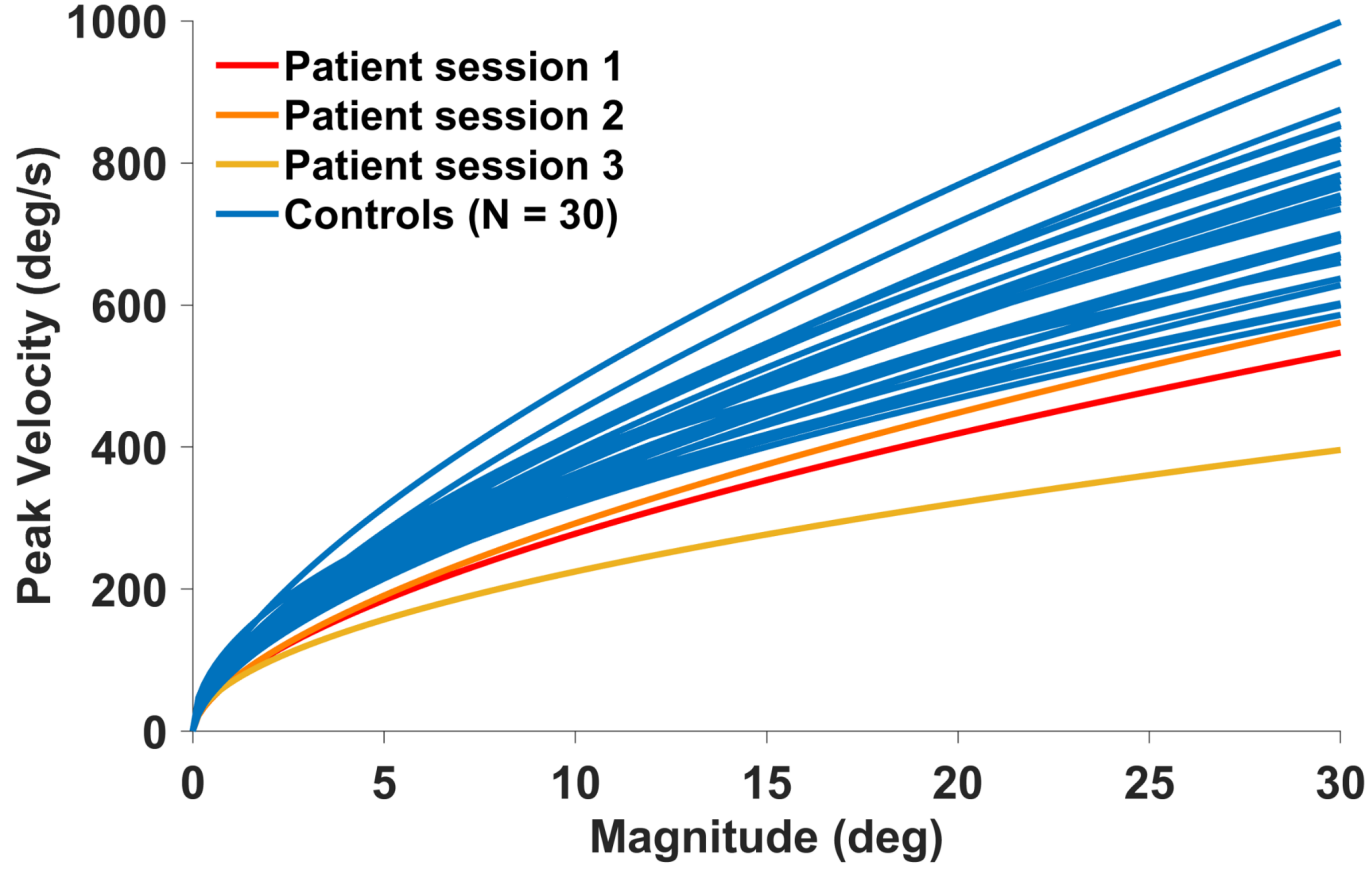
Main sequence for all the oculomotor tasks combined. The patient data (sessions 1 through 3) are displayed in warm colors; the control participants data is shown in blue. See SI Figure 5 for a visualization of each saccade contributing to these curves.

We analyzed the slopes of the relationship between saccadic magnitude and saccadic peak velocity, *i.e.* the saccadic main sequence, to investigate potential deficits in saccadic dynamics. To account for the unequal N when testing for significance, we conducted an Earth Mover’s Distance analysis (a metric which measures the “distance” between pairs of distributions) to measure the difference between control participants and each other control (as a baseline), as well as the difference between each session from the patient and the control data. We then conducted a Mann-Whitney U test (comparing the median distance between controls and other controls and the median distance between the patient and controls). The data revealed decreased saccadic peak velocities (*p*<.000001), and consequently, lower main sequence slopes, for the patient (in each of the three testing sessions) relative to the control participants (Figure 5).

#### 2.5.3 Free viewing

**Figure 6.**
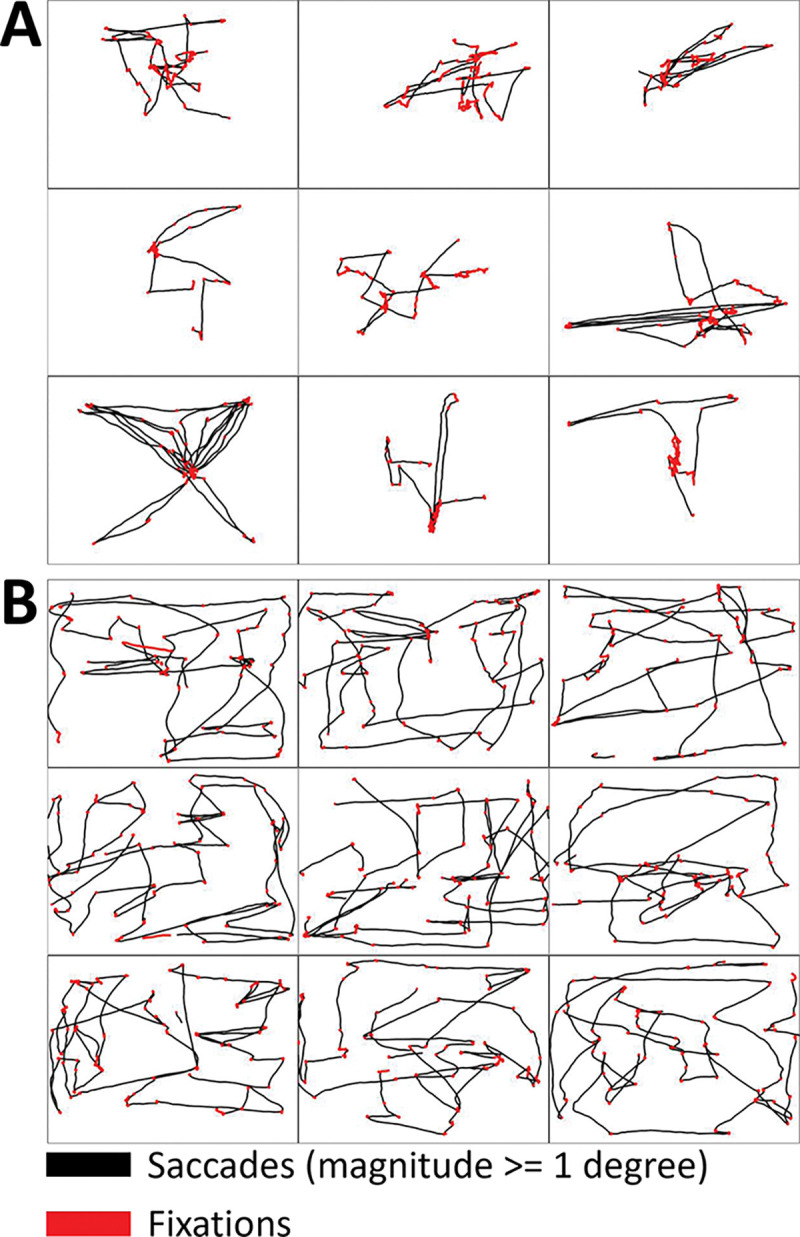
Scanpaths during free viewing of 9 individual natural images. **(A)** Scanpaths from the patient’s first testing session. **(B)** Scanpaths from a representative control participant. Fixations are indicated in red.

The patient’s gaze positions, during free visual exploration of natural scenes, were constrained to a more reduced area of visual space than those of control participants, and presented a much stronger central bias. The patient’s effective area of exploration, as evidenced by scanpaths, was more dramatically diminished in the first testing session (Figure 6) than in testing sessions 2 and 3 (**SI Figure 4)**.

### 2.6. Neuroimaging

**Figure 7.**
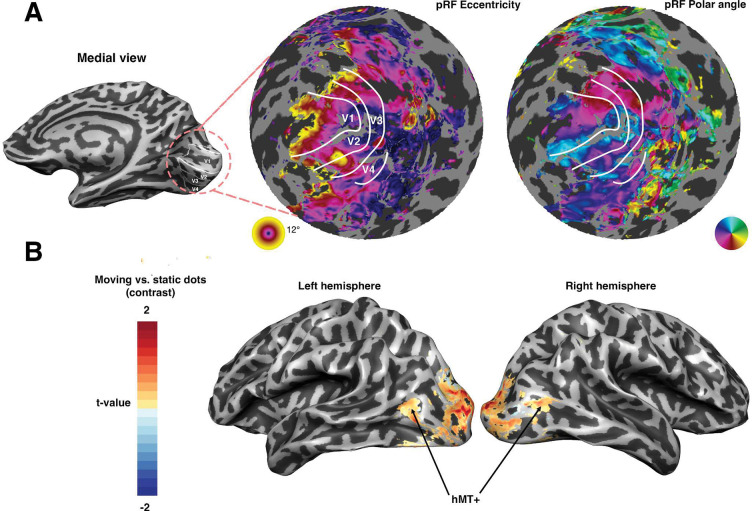
Population receptive field (pRF) mapping and motion localizer on the cortical surface. **(A)** Right hemisphere with an overlay of pRF eccentricity (middle) and polar angle (right) in early visual areas. White contours indicate boundaries between visual maps. Both maps reveal orderly retinotopic organization with upper and lower visual field representations and eccentricity increasing from posterior to more anterior parts of the surface. The inset on the left shows the anatomical location of the early visual cortex on the medial inflated surface. **(B)** Lateral view of the same hemisphere showing activation for the motion localizer contrast (moving vs. static dots). Color scale indicates t-values, with warm colors representing stronger responses to motion. Significant motion-selective responses are observed in area MT

To identify the neural correlates of the perceptual deficit, we conducted two fMRI experiments. First, we employed population receptive field (pRF) mapping to examine the spatial organization of visual responses across the cortex. This revealed typical retinotopic maps in early visual areas (V1, V2, V3, and V4), indicating intact visual field representations (Figure 7A).

To probe motion-specific responses, we conducted a localizer experiment targeting motion responsive area MT. Responses in MT were robust (Figure 7B) and did not obviously differ from those observed in neurotypical individuals (Amano et al., 2009; Huk et al., 2002). This suggests that the motion-selective cortex was functionally intact in the patient, and that the deficit might be related to impaired integration or access to motion signals, or some other cortical deficit, rather than a loss of basic motion sensitivity.

**Figure 8.**
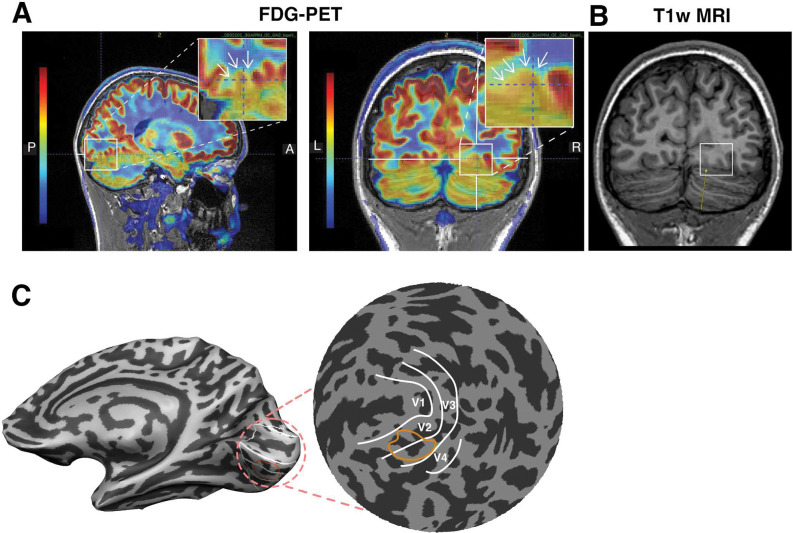
FDG-PET showing reduced glucose metabolism in pRF-defined regions of the right ventral visual cortex. **(A)** Sagittal and coronal slices of the FDG-PET map. Insets highlight the lingual gyrus, with arrows indicating areas of hypometabolism. **(B)** The same coronal slice of a T1-weighted image shows cortical gray/white matter blurring (white rectangle). **(C)** Right hemisphere cortical surface view with a zoomed-in inset of the occipital lobe. White lines delineate visual field map boundaries, and the orange outline indicates the hypometabolism on the cortical surface.

Finally, the patient underwent an exam involving FDG PET imaging, which revealed a subtle posterior right lingual gyrus cortical signal abnormality with corresponding severe focal FDG hypometabolism, suggestive of focal cortical dysplasia (Figure 8A). The same region on a T1-weighted image showed cortical gray/white matter blurring (Figure 8B). Increased gray/white matter blurring in focal cortical dysplasia has been associated with increased behavioral impairments (Blackmon et al., 2015). This exam also revealed a symmetric moderate bilateral occipital and medial temporal lobe FDG hypometabolism consistent with a temporal occipital seizure network.

We projected the location of the focal FDG hypometabolism in the posterior right lingual gyrus back onto the cortical surface (Figure 8C). This suggests that the deficit can be localized to the ventral aspect of V2 and V3. Subsequent inspection of the retinotopic maps in Figure 7 reveals abnormally large receptive field sizes in that location. It is tempting to speculate that this abnormality is related to the FDG and contributes to the perceptual deficits experienced by the patient, although there are no clear reported cases of the impact of such an abnormality in the literature.

## 3. Discussion

This case study presented a unique instance of sudden-onset, persistent visual impairments in a 12-year-old male, characterized by subjective akinetopsia-like symptoms despite normal motion perception as assessed by standard psychophysical testing. The key findings were a significant impairment in object recognition within clutter, both in moving and static displays, and atypical oculomotor kinematics, coupled with a focal cortical abnormality in the right lingual gyrus, as revealed by PET imaging. These results challenge the traditional view that reports of akinetopsia should always be understood as a deficit in motion processing, suggesting that higher-level visual processing, particularly object recognition in cluttered environments, can manifest as subjective motion perception difficulties.

The patient’s performance on random dot motion tasks indicates that his primary visual motion processing pathways, including MT/V5, were likely intact based on studies in macaque (Newsome & Paré, 1988) and human (Rees et al., 2000). This interpretation is supported by the absence of PET and MRI findings in dorso-lateral cortex, and by normal fMRI responses to visual motion in lateral temporal cortex. Instead, the significant deficits in object recognition in clutter are more consistent with dysfunction in the ventral visual stream (Goodale & Milner, 1992; Ungerleider & Haxby, 1994), or in communication between ventral and dorsal areas. Consistent with the ventral stream interpretation, MRI revealed blurring of gray-white contrast in the right mid lingual gyrus extending to the inferior temporal gyrus with concordant FDG PET hypometabolism, suggestive of focal cortical dysplasia (FCD). FCD, a common cause of drug-resistant epilepsy, can lead to diverse neurological deficits depending on its location and extent (Bast, 2008). Although cortical dysplasia is a developmental malformation, the consequences may not become apparent until later in adolescence, for example in late onset epilepsy (Bartolomei et al., 1999). In this case, the localized FCD in the lingual gyrus may have disrupted the patient’s ability to segregate and recognize objects in cluttered scenes, leading to the subjective experience of motion blindness, especially in complex dynamic environments.

The ventral visual stream interpretation is complicated, however, by the patient’s atypical smooth pursuit and saccadic eye movements. Eye movement deficits are more typically associated with lesions to cerebellum, parietal or frontal cortex, or subcortical nuclei (Sharpe, 2008). However, the ventral occipital site of the hypometabolism is connected to dorsal visual areas via the vertical occipital fasciculus, the major pathway for communication between ventral and dorsal visual streams in human and other primates (Takemura et al., 2016; Yeatman et al., 2014). This pathway is thought to be crucial for the exchange of visual information concerning object properties (form, color, identity) and spatial information. If a ventral occipital FCD altered physiology, for example triggering slow waves of Leão or subclinical seizures, then symptoms might result from altered activity in regions beyond what is visible in the PET images, including neighboring cortical tissue and more remote areas connected by white matter tracts. More generally, smooth pursuit is affected not only by motion signals, but also by visual attention (Ferrera & Lisberger, 1995) and cognition (Kowler, 1989), and its impairment could be a secondary consequence of the recognition deficit. The inability to quickly and accurately identify moving objects in clutter would naturally hinder the ability to smoothly track them. We also cannot rule out the opposite causality, namely that an oculomotor deficit is primary, giving rise to perceptual deficits. This alternative, however, would not explain the patient’s deficit in recognition of static objects in clutter.

The discrepancy between subjective symptoms and psychophysical findings highlights the importance of considering higher-level cognitive processes in the interpretation of visual perception deficits. A similar issue occurs with cortical visual impairment (CVI), in which subjective deficits are often not fully captured by basic visual measurements such as acuity, perimetry and contrast sensitivity (C. Williams et al., 2021); rather, the subjective deficits may be better reflected in higher level or more naturalistic tasks such as visual search (Manley et al., 2022). This case differs from typical CVI, however, in that CVI is typically associated with a known etiology (such as perinatal ischemia) and clear neurological deficits (Huo et al., 1999).

The patient’s experience of “things suddenly appearing” suggests that his visual system was struggling to integrate and interpret dynamic visual information, particularly in cluttered scenes. This interpretation aligns with the concept of “crowding,” where the presence of surrounding stimuli interferes with the recognition of a target object (Whitney & Levi, 2011). In this case, the patient’s crowding distance was significantly increased, indicating a profound impairment in object recognition in clutter.

Several limitations should be considered. First, this is a case study, and the findings may not be generalizable to all patients with similar symptoms. Second, while PET imaging revealed a focal abnormality, the precise nature of the FCD and its functional impact remain to be fully elucidated. Third, the long-term effects of this condition are yet unknown.

Future research should explore the neural mechanisms underlying object recognition in clutter and its relationship to motion perception. Investigating the role of the ventral occipital cortex in dynamic scene analysis – the interplay between the ventral and dorsal visual streams in complex visual tasks would be particularly valuable. Additionally, exploring the potential for rehabilitation strategies targeting object recognition in clutter could have significant clinical implications for patients with similar visual impairments.

In conclusion, this case study provides compelling evidence that subjective akinetopsia-like symptoms can arise from deficits in object recognition in clutter, and without apparent trauma or other illness. The deficit may be mediated by physiological changes induced by focal cortical dysplasia in the ventral temporal visual cortex. It underscores the importance of a comprehensive assessment of visual function, including higher-level cognitive processes, in patients presenting with atypical visual symptoms.

## 4. Methods

### 4.1. General Methods

All testing procedures at New York University (including functional and anatomical MRI were approved by the New York University Institutional Review Board prior to participation. Written informed consent was obtained from each participant, as well as written parental consent from participants under the age of 18, and written assent for participants under the age of 13.

At SUNY Downstate, this case study was reviewed by the Institutional Review Board and was declared “not research involving human subjects” based on the definitions as defined by applicable U.S. federal regulations. For control participants, procedures at SUNY Downstate were carried out under the guidelines and approval of the SUNY Downstate Institutional Review Board (protocol number 690152). Written informed consent was obtained from each control participant, as well as written parental consent from control participants under the age of 18 and written assent for control participants under the age of 13.

### 4.2. Magnetic Resonance Imaging

#### 4.2.1 Acquisition and preprocessing

Structural and functional data were acquired from the patient on a 3 T Siemens MAGNETOM Prisma MRI scanner (Siemens Medical Solutions, Erlangen, Germany) at the Center for Brain Imaging at NYU. Both the structural and functional images were acquired using a Siemens 64-channel head coil. MPRAGE anatomical images were acquired (TR, 2400 ms; TE, 2.4 ms; voxel size, 0.8mm3 isotropic; flip angle, 8°) and auto-aligned to a template. Across two sessions, nine 5-minute functional echo-planar images (EPIs) were collected using a CMRR T2*-weighted MulitBand EPI sequence (Feinberg et al., 2010; Moeller et al., 2010; Xu et al., 2013). Six of the EPIs were for measuring retinotopic maps and 3 for localizing motion responses. The parameters of the EPI scans were as follows: repetition time (TR) of 1000 ms, echo time (TE) of 37 ms, voxel size of 2 mm^3^, flip angle of 68°, multiband acceleration factor of 6, and posterior-to-anterior phase encoding. Additionally, two distortion maps were acquired per session to correct susceptibility distortions in the functional images: one spin-echo image with anterior-posterior (AP) and one with posterior-anterior (PA) phase encoding. Anatomical and functional preprocessing was performed using fMRIPrep v.23.1.2

#### 4.2.2 Retinotopy

In the retinotopic mapping scans, a colorful contrast pattern was viewed through a moving aperture, either in the shape of a sweeping bar (three 5-minute scans) or a combination of rotating wedge and expanding or contracting ring (three 5-minute scans), as described in prior work (Himmelberg et al., 2025; Kurzawski et al., 2025). The stimuli were limited to a circular window centered at a fixation cross, 12.2° radius. The retinotopy scans were analyzed by fitting each voxel’s time series with a population receptive field model, a 2D circular Gaussian parameterized by its location in the visual field (x,y) and size (Gaussian SD). The data were analyzed using vistasoft software (https://github.com/vistalab/vistasoft), as described previously ((Himmelberg et al., 2025; Kurzawski et al., 2025).

#### 4.2.3 Motion Localizer

The motion localizer consisted of white dots moving radially (inward and outward) at three eccentricities: centered on fixation and at 10° along the left and right horizontal meridians. Dots were presented on a black background and confined to circular apertures with a 4° radius. Each condition lasted 12 seconds, followed by a 12-second blank, forming 36-second blocks. Each run included 8 such blocks, ending with an additional 12-second blank. Dot motion was scaled according to eccentricity, such that motion was slow near the center and faster toward the aperture edge.

#### 4.2.4 Visualization

Volumetric maps were visualized in iTKsnap (Yushkevich et al., 2006) and cortical meshes were rendered in BrainVoyager 23.2 (Goebel, 2012).

### 4.3. Psychophysics

An Apple iMac 27″ computer presented stimuli on an external monitor, LG 27” UltraFine 5K (27MD5KL-B), with a 59.5 × 33.5 cm screen with 5,120 × 2,880 pixels, and a frame rate of 60 Hz. The maximum luminance was about 275 cd/m^2^. The participants placed their chin on an adjustable chin rest 40 cm away from the monitor, and used either a keyboard (random dot motion) or verbal response (letters in noise and crowding) to perform the tasks. In the latter case, the experimenter (author J.K.) recorded the responses without being able to see the display.

#### 4.3.1 Random dot motion

128 black and white dots were presented within a large (18 deg radius) mid-gray aperture. All coherent motion dots moved in the same direction, either left or right. Each non-coherent dot moved in a random direction sampled from a uniform 0–360 deg distribution. All dots had a 50 ms lifetime, and were replaced with a dot at a new random location within the aperture when it reached the end of its life. Dots that moved beyond the edge of the aperture reappeared at the opposite edge. A dot was presented in the center of the aperture to aid fixation. Participants judged stimulus motion direction (left or rightward) by pressing the left/right key on a keyboard.

In three motion-discrimination experiments we varied stimulus speed, duration, and coherence respectively. Values for stimulus properties not manipulated in a particular experiment were set to 8 deg/sec for stimulus speed, 500 ms for stimulus duration, and both 25 and 100% for stimulus coherence. These sets of parameters swept out the typical space of greatest variability in motion sensitivity. In each experiment each combination of stimulus coherence, speed, and duration was repeated 30 times.

We performed these experiments with the patient, as well as an age- and sex-matched control (C1).

#### 4.3.2 Letters in noise

Participants performed a letter identification task designed to assess recognition in cluttered visual environments. Each trial began with a fixation period, during which participants maintained gaze on a central red dot. Following fixation, a single target letter moved horizontally for 1 second across the screen, either to the left or right, at a constant speed of 4 deg/sec. The direction of motion was randomized across trials. Once the letter started moving participants were able to move their gaze. Throughout the experiment, a variable number of distractor letters remained visible. Three levels of visual clutter were defined by the total number of distractor letters randomly distributed across the screen: 30, 600, or 1000. Distractors were sampled from a fixed set of 9 letters (Z, H, V, R, K, H, C, X), randomly rotated, and spatially jittered across trials with no constraints apart from full-screen coverage. Letters were rendered in Technology font (https://www.dafont.com/technology-2.font). There were 60 trials per clutter level. At the end of each trial, participants were asked to report the identity and direction of the target letter. We also performed a letter identification task in the fovea where the target letter was presented in the fovea and the background distractors moved either left or right. In this case participants were asked to only identify the letter. All stimuli were viewed at a distance of 40 cm. We performed the letters in noise experiments with the patient, as well as an age- and sex-matched control (C1).

#### 4.3.3 Measuring crowding and acuity

Acuity and crowding were measured following the procedure described in Kurzawski et al. (2023). In short, to assess acuity, we presented isolated letters and varied their size. To assess crowding, we presented trigrams of three adjacent letters arranged radially around fixation and varied the spacing between letters. We used the same set of 9 letters (Z, H, V, R, K, H, C, X) rendered in Sloan font (Pelli et al., 1988; Sloan, 1959). Acuity was measured both at the fovea and at 5 degrees eccentricity along the left and right horizontal meridians, while crowding was tested only in the periphery at the same locations. Each trial began with a central fixation cross. After 250 milliseconds of stable fixation, the stimulus was presented for 150 milliseconds at a fixed eccentricity. In crowding trials, the target was the middle letter of the trigram. Observers identified the target by clicking on a response screen displaying all possible letters. In acuity trials there was only one letter presented. Correct responses were followed by a brief auditory tone. The next trial began only after participants maintained gaze within 1.5 degrees of fixation for at least 250 milliseconds. If this requirement was not met within ten seconds, the system prompted eye tracker recalibration. Thresholds were measured using Quest staircases. Measurements of peripheral acuity for adult control participants did not include gaze tracking, as these sessions were conducted before eye tracking was available in the lab.

### 4.4. Oculomotor Methods

#### 4.4.1 Participants

Control participants (17 female, 13 male) ranged in age from 11 to 55 (median 23 years). The patient underwent oculomotor testing on three occasions over one year from late 2022 to 2023. Each control participant was tested in a single session.

#### 4.4.2 Stimuli and Procedure

Participants rested their forehead and chin on the EyeLink 1000 head/chin support, ~57 cm away from a linearized video monitor (Barco Reference Calibrator V, 60 Hz refresh rate).

Participants’ eye movement and gaze kinematics were assessed with an established battery designed to sample different dimensions of oculomotor behavior (Brunet et al., 2024). The battery, which took about 25 minutes to complete, consisted of eight visual tasks: Fixation, Saccade Speed, Saccade Accuracy, Visually Guided, Anti-Saccade, Memory Guided, Free-Viewing, and Smooth Pursuit.

The Fixation Task was used to assess blinks, saccadic intrusions, and fixation instability (as indicated by the dispersion in eye positions) during attempted fixation. A single fixation target was displayed: first at the center of the screen, then randomly either 20° to the left or right, or 15° up or down. The target then appeared in the center again. The target was displayed for 30 seconds at each location. Participants were instructed to keep their gaze on the target as accurately as possible. This sequence was repeated six times.

In the Saccade Speed Task, participants were instructed to switch their gaze back and forth as quickly as possible between two targets, which were presented 15° or 20° apart, either horizontally or vertically (one horizontal and one vertical trial, in random order). Each of the two trials was 50 seconds long. This task was used to assess saccadic latency, saccadic magnitude and peak velocity (and main sequence slopes), and saccadic targeting errors.

The Visually Guided Task was a variant of a prosaccade task (Hutton, 2008) in which the fixation target was initially displayed at the center for 1 sec and then moved to a single point at 20° eccentricity to the right or left. Participants were instructed to keep their gaze on the target, moving their eyes as quickly and as accurately as possible. After an inter-trial interval of 2500–3500 ms the next trial began, and this sequence continued for two minutes (for a total of 30 trials).

The Anti-Saccade Task followed the same sequence as described above, but participants were instructed to maintain fixation until the target moved to an eccentric position, and then move their eyes to the equivalent location on the side of the screen opposite to the target.

In the Saccade Accuracy Task, the target was first presented on the center of the display and then moved in an alternating sequence to increasingly distant locations on the left or right side of the display (or, on vertical trials, on the top or bottom of the display), and then returned in a reverse sequence to the center of the display. For example, in one sequence the target moved from 0° to 5°, −5°, 10°, −10°, 15°, 15°, 20°, −15°, 15°, −10°, 10°, −5°, 5°, and then to 0° degrees of visual angle. Vertical trials extended only to 15°, and participants saw one horizontal and one vertical trial per session. Participants were instructed to keep their gaze on the moving target, and to do so as accurately as possible.

In the Memory-Guided Task, a target appeared at either 10°, 12.5°, 15° 17.5° or 20°, to the left or the right of the center, while the participant maintained central fixation. After 100 msec, this peripheral visual target disappeared. After a further 500 ms delay, the central fixation target disappeared. Participants were instructed to maintain central fixation until the central fixation target disappeared, and then to make an eye movement as quickly as possible to the remembered location of the peripheral visual target. This sequence continued for 30 trials per session. The primary difference between the Memory-Guided Task and the Visually Guided Task is that the Memory-Guided Task included a temporal delay before a saccade was launched (Hikosaka & Wurtz, 1983).

In the Free-Viewing Task, participants saw 9 different natural images (from (Otero-Millan et al., 2008) in random order, with each image being presented for 20 seconds. Participants were instructed to freely look around the images in any way they liked.

In the Smooth Pursuit Task, participants were instructed to follow, as accurately as possible, a target that moved sinusoidally along the horizontal meridian, with an amplitude of 7.5° and a frequency increasing from 0.5 Hz to 3 Hz (with frequency increments of 0.5 Hz every 10 seconds, ending after 60 seconds). All six target speeds repeated twice each session.

Some of the present analyses include data compiled from all the above tasks, whereas other analyses are limited to data from the Smooth Pursuit Task and the Free-Viewing Task (as these two specific tasks revealed the patient’s most notable oculomotor deficits).

#### 4.4.3 Eye Movement Recording and Analysis

Gaze position was recorded using an EyeLink 1000 Plus eye tracker sampling at 500 Hz. We identified and removed blink periods as portions of the raw data where pupil information was missing. We also removed portions of data where very fast decreases and increases in pupil area occurred (50 units/sample; such periods are probably partial blinks where the pupil is never fully occluded) (Troncoso et al., 2008). We added 200 ms before and after each blink or partial blink to eliminate the initial and final parts where the pupil was partially occluded (Troncoso et al., 2008). We detected saccades with a modified version of the algorithm that was developed by (Engbert & Kliegl, 2003, 2004) and implemented in a number of recent studies (Alexander, Mintz, et al., 2021; Alexander et al., 2024; Alexander, Venkatakrishnan, et al., 2021; Otero-Millan et al., 2020), using a minimum saccadic duration of 6 ms and λ = 6. To reduce the amount of potential noise, we considered only binocular saccades, that is, saccades with a minimum overlap of one data sample in both eyes (Engbert, 2006; Engbert & Mergenthaler, 2006; Laubrock et al., 2005; Rolfs et al., 2006). Additionally, we imposed a minimum intersaccadic interval of 20 ms so that potential overshoot corrections might not be categorized as new saccades (Møller et al., 2002). Further details about our eye movement analyses are extensively described in previous publications (McCamy et al., 2012). Microsaccades were defined as saccades with magnitude < 1° in both eyes. This 1° cutoff captures the distribution of fixational microsaccades made in most contexts (e.g. (Martinez-Conde et al., 2013; Rolfs, 2009). See (Alexander & Martinez-Conde, 2019) for further practical detail of these microsaccade analysis methods. Fixation periods (i.e. during free viewing) were defined as periods of relative gaze stability between large (> 1°) saccades. As such, fixation periods could potentially include both fixational drift and microsaccades.

##### Smooth pursuit performance analysis.

To quantify smooth pursuit performance, we analyzed the gain, catch-up saccade production, and error. Gain was defined as the ratio of the velocity of the target to the velocity of the eye, indicating how accurately the eye matches both the speed and direction of the target. Values close to 1 indicate a good match; values larger than 1 indicate that the eye was moving faster than the target; and values less than 1 indicate that the eye was moving slower than the target. Catch-up saccades refer to the small saccades produced when smooth pursuit performance deteriorates and gaze position lags behind the target too much, in order to compensate for this error (Barnes, 2008). Error was defined as the difference between the speed of the eye and the target, without regard to direction.

##### Free-viewing analysis.

We recorded scanpaths for each of the 9 natural images viewed by each participant. We note that different participants may have viewed different 9-image sets, with some of the images overlapping across sets and participants, and others not. This was also true for the patient’s different recording sessions. Moreover, our software at the time did not allow us to match specific scanpaths to specific images. Even so, the overall scanpath patterns (irrespective of their corresponding images) reveal striking differences between the patient’s free-viewing performance and that of control participants, especially for the patient’s initial recording session (see Figure 6).

##### Main sequence analysis.

We assumed a power-law relationship between saccade magnitude and saccade peak velocity. We assumed a power-law relationship rather than a linear one because the r^2^ was always higher for the linear fits of the log-transformed data than for linear fits of the raw data. Thus, we performed robust linear regressions (using the robustfit function in MATLAB) on the log-transformed data for each subject to obtain the slope for each main sequence relationship.

## Supplementary Material

Supplementary Files

This is a list of supplementary files associated with this preprint. Click to download.

• Sp2SubjCond123TimealignedTest2.mp4

• Sp2SubjCond124TimealignedTest2.mp4

• Sp2SubjCond124TimealignedTest3.mp4

• SuppMovieCaptionsOct112025.docx

• Sp2SubjCond123TimealignedTest3.mp4

## Significance Statement

A 12-year-old experienced sudden, akinetopsia-like visual impairment. However, subsequent testing revealed that the core deficit was object recognition in clutter, not motion perception *per se*. Neuroimaging showed a focal cortical abnormality in the right posterior occipital cortex. This suggests localized higher-level visual processing dysfunction can mimic motion perception difficulties, highlighting the need for thorough assessment in pediatric visual impairments.
